# Impact of Cerebral Embolic Protection on Cognitive Function After Transcatheter Aortic Valve Implantation: Data From the BHF PROTECT-TAVI Randomized Trial

**DOI:** 10.1161/CIRCULATIONAHA.125.076761

**Published:** 2025-08-30

**Authors:** James Kennedy, Daniel J. Blackman, Matthew Dodd, Anna Poggesi, Laura Read, Zahra Jamal, Richard Evans, Tim Clayton, Rajesh K. Kharbanda, David Hildick-Smith

**Affiliations:** Radcliffe Department of Medicine, University of Oxford, Oxford, UK (J.K., R.K.K.).; National Institute for Health and Care Research Oxford Biomedical Research Centre, Oxford University Hospitals NHS Foundation Trust, Oxford, UK (J.K., R.K.K.).; Department of Cardiology, Leeds Teaching Hospitals NHS Trust, Leeds, UK (D.J.B.).; Clinical Trials Unit, Department of Medical Statistics, London School of Hygiene and Tropical Medicine, London, UK (M.D., L.R., Z.J., R.E., T.C.).; NEUROFARBA Department, University of Florence, Florence, Italy (A.P.).; Department of Cardiovascular Medicine, John Radcliffe Hospital, Oxford, UK (R.K.K.).; Sussex Cardiac Centre, University Hospitals Sussex, Brighton, UK (D.H.-S.).

**Keywords:** cognition, embolic protection devices, transcatheter aortic valve replacement

## Abstract

**BACKGROUND:**

In addition to the risk of stroke, patients undergoing transcatheter aortic valve implantation (TAVI) are susceptible to a decline of neurocognitive function. This may occur because of embolization of material (eg, valve or calcium) to the brain. Cerebral embolic protection (CEP) devices are engineered to capture this debris, potentially mitigating its incidence.

**METHODS:**

This is a secondary analysis of the BHF PROTECT-TAVI trial (British Heart Foundation Randomized Trial of Routine Cerebral Embolic Protection in Transcatheter Aortic Valve Implantation), in which participants with aortic stenosis from across 33 centers in the United Kingdom were randomly assigned at a 1:1 ratio to undergo TAVI with a CEP device (SENTINEL, Boston Scientific; SENTINEL CEP group) or TAVI without a CEP device (control group). This analysis is restricted to those who underwent cognitive assessment. The primary outcome was the mean change in the telephone version of the Montreal Cognitive Assessment (t-MoCA) between baseline and 6 to 8 weeks after TAVI. The secondary outcome was a ≥3-point drop in total t-MoCA score between baseline and 6 to 8 weeks after TAVI.

**RESULTS:**

A total of 3535 participants, 1763 in the SENTINEL CEP group and 1772 in the control group (mean age 81.0 years, 37.7% women) randomized in BHF PROTECT-TAVI were included in the modified intention-to-treat population for this analysis. The median t-MoCA at presentation was 18 (interquartile range, 16–20). The median t-MoCA at 6 to 8 weeks was 20 (interquartile range, 17–21). The mean change in total t-MoCA score between baseline and 6 to 8 weeks adjusted for the baseline score was 0.83 (95% CI, 0.70–0.96) in the SENTINEL CEP group and 0.91 (95% CI, 0.79–1.04) in the control group. There was no difference in means between the treatment groups (−0.07 [95% CI, −0.22 to 0.09], *P*=0.42). The incidence of a ≥3-point drop in the total t-MoCA score was 154 of 1763 (8.7%) in the SENTINEL CEP group and 142 of 1772 (8.0%) in the control group. The corresponding risk difference was 0.72% (95% CI, −1.10 to 2.55; *P*=0.44). These findings were robust to sensitivity analyses. There was no evidence of an interaction between treatment assignment and any of the subgroups assessed.

**CONCLUSIONS:**

In the BHF PROTECT-TAVI trial, the use of CEP did not impact cognition after TAVI.

**REGISTRATION:**

URL: https://www.isrctn.com; Unique identifier: ISRCTN16665769.

**Clinical Perspective**

**What Is New?**

- In the overall population, cognitive function measured using the telephone version of the Montreal Cognitive Assessment showed a small increase, on average, 6 to 8 weeks after transcatheter aortic valve implantation.
- Approximately 1 in 10 patients showed a reduction of ≥3 points on the telephone version of the Montreal Cognitive Assessment after transcatheter aortic valve implantation.
- Cerebral embolic protection had no impact on the changes in cognitive function after transcatheter aortic valve implantation.

**What Are the Clinical Implications?**

- The routine use of cerebral embolic protection does not improve cognitive function 6 to 8 weeks after transcatheter aortic valve implantation.
- Further work is needed to understand the impact of cognitive function on clinical outcomes.

**Editorial, see p 1279**

Postoperative cognitive dysfunction has long been acknowledged as a complication of cardiac surgery.^1,2^ As transcatheter aortic valve implantation (TAVI) is increasingly used as a treatment for patients with severe aortic stenosis, so efforts to understand the incidence, risk factors, and longer-term consequences of cognitive changes have increased.^3^

Two large randomized trials enrolling a combined total of >10 000 participants have recently investigated the routine use of cerebral embolic protection (CEP) at the time of TAVI. Neither the PROTECTED TAVR nor the BHF PROTECT-TAVI (British Heart Foundation Randomized Trial of Routine Cerebral Embolic Protection in Transcatheter Aortic Valve Implantation) study demonstrated a reduction in the incidence of stroke at 72 hours (or discharge if sooner), the primary outcome of each trial.^4,5^ In addition to the risk of stroke, CEP devices may mitigate the risk of a decline in neurocognitive function by capturing embolized debris (eg, valve or calcium). In this secondary analysis of the BHF PROTECT-TAVI trial, we evaluated the change in cognition using the Montreal Cognitive Assessment (MoCA)^6^ from baseline to 6 to 8 weeks after TAVI with CEP use.

## METHODS

### Trial Design and Oversight

This is a secondary analysis of the BHF PROTECT-TAVI trial, a UK-based prospective, open-label, blinded, outcome-adjudicated, multicenter, superiority parallel group, randomized, controlled trial. The BHF PROTECT-TAVI (registered on ISRCTN on June 23, 2020; URL: https://www.isrctn.com/ISRCTN16665769; unique identifier: ISRCTN16665769) trial design, analysis plan, and main results have been published previously.^4,7^ The trial evaluated the routine use of the SENTINEL CEP device (Boston Scientific) to prevent stroke in participants with aortic valve stenosis undergoing TAVI. The trial was investigator initiated and approved by the UK Health Research Authority (REC 20/WA/0121, IRAS276396). The British Heart Foundation (BHF clinical study No. CS/20/1/34732) funded the trial. Boston Scientific provided the SENTINEL CEP devices through an investigator-sponsored research grant (ISRCAR00332) but was not involved in the design, conduct, or reporting of the trial. An independent trial steering committee (TSC) and data monitoring committee (DMC) oversaw the trial. An independent clinical events committee blinded to treatment allocation adjudicated the main trial’s primary outcome of stroke. Patients were involved in the design of the trial, and 2 patient representatives served as independent members of the TSC. The trial was sponsored by University of Oxford. The London School of Hygiene and Tropical Medicine Clinical Trials Unit coordinated the trial and performed the statistical analyses. The first authors drafted this manuscript, with all authors contributing to revisions and approving the submission for publication.

### Participants

We enrolled trial participants with aortic stenosis who were scheduled to undergo TAVI and, in the opinion of the treating physician, were clinically and anatomically suitable for treatment with SENTINEL CEP. All participants were ≥18 years of age and provided written informed consent to participate in the trial. There were no specific exclusion criteria. The participants were randomly assigned at a 1:1 ratio to undergo TAVI with SENTINEL CEP (CEP group) or without SENTINEL CEP (control group). Randomization was stratified according to trial site with the use of random permuted blocks (sizes 4 and 6). An independent statistician from Sealed Envelope Ltd (UK) prepared the randomization codes, and randomization was done via the secure Sealed Envelope website.

### Trial Procedures

The SENTINEL CEP device is usually delivered from the right radial artery and deploys filters in the left common carotid (distal filter) and right innominate (proximal filter) arteries. There was no mandated screening of the aortic arch anatomy, and participant selection was left to the discretion of the treating physician. Full deployment of the SENTINEL CEP device was defined as correct placement of both filters for the duration of the TAVI procedure.

Participants underwent cognitive assessments using the MoCA^6^ before TAVI, 72 hours after TAVI (or before discharge if sooner), and at 6 to 8 weeks after TAVI. Given that no face-to-face assessments were mandated in the protocol after discharge, use of the full MoCA and the telephone version of the MoCA (t-MoCA)was allowed to complete the cognitive assessment at 6 to 8 weeks. The full MoCA is composed of 7 different scored components to test cognition with a maximum score of 30: visuospatial/executive (maximum 5 marks), naming (3 marks), attention (6 marks), language (3 marks), abstraction (2 marks), delayed recall/memory (5 marks), and orientation (6 marks). The t-MoCA is identical, but the visuospatial/executive and naming components are omitted, leaving a maximum possible score of 22. To allow the comparison of cognitive assessments between baseline and 6 to 8 weeks, only the t-MoCA scores were used. If the full MoCA score was available, then the scores for the visuospatial/executive and naming components were removed from the total. The same English version of the MoCA was used at all time points.

The case report form required entry of the blood pressure immediately before TAVI and the lowest blood pressure measured during the TAVI procedure.

### Trial Outcomes

The primary outcome for this secondary analysis was the change in the total t-MoCA score between baseline and 6 to 8 weeks after TAVI. In addition, a secondary outcome, consisting of a drop in the total t-MoCA of ≥3 between baseline and 6 to 8 weeks, was assessed.^8^

Periprocedural hypotension was defined in 2 ways: absolute, in which the lowest periprocedural blood pressure dropped below a mean arterial pressure of 70 mm Hg, and relative, in which the threshold to demonstrate a drop in blood pressure was adjusted for the preprocedural blood pressure measurement.^9^

### Statistical Analysis

BHF PROTECT-TAVI had a calculated sample size of 7730 participants that would have 80% power at a 5% significance level to show the superiority of SENTINEL CEP if the incidence of stroke by 72 hours (or hospital discharge if discharge was sooner) was 3% in the control group and 2% in the CEP group, allowing for a 1% loss to follow-up. After a third interim analysis, the data monitoring committee recommended to the TSC that the trial discontinue enrollment, as the prespecified futility criterion had been met. Enrollment was discontinued on October 9, 2024, after the randomization of 7635 participants. The trial is expected to complete follow-up in November 2025.

During the trial, it became clear to the Trial Management Group that the collection of multiple MoCA assessments for each participant was resource intensive, and, to maximize recruitment for the primary outcome analysis, a proposal to stop collection of MoCA data was developed on the basis of blinded data from 4701 participants randomized up to July 21, 2023. Given that the primary outcome analysis was powered to detect differences in stroke incidence and based on low events rates, for continuous MoCA outcomes, BHF PROTECT-TAVI would provide additional power to detect even small differences between trial arms. Power calculations were submitted to the TSC based on these data, including the combined SDs at each time point and correlations between time points (Supplemental Material). As an example, a study population of 390 participants would give 90% power to detect a difference of 1 point on the MoCA with a conservative SD of 3.5 and correlation of 0.5 between baseline and follow-up. The proposal was accepted by the TSC, the trial protocol was amended, and MoCA data collection was stopped.

To be eligible for this analysis, the due date for the 6- to 8-week follow-up had to be before January 4, 2024, the date when the protocol amendment was issued.

The primary and secondary cognitive outcome measures were assessed on the modified intention-to-treat (ITT) population. The modified ITT population included all randomized participants whose TAVI procedure was started, according to the group to which they were assigned, irrespective of whether they received the intervention as intended. The participant had to have a MoCA performed at baseline and at 6 to 8 weeks to be included in the modified ITT population. The TAVI procedure was considered to have started when the first arterial puncture was performed. The primary outcome was assessed using an analysis of covariance model adjusting for baseline t-MoCA scores. For the secondary outcome, risk differences were calculated using generalized linear models for binomial outcomes with an identity link function and without adjustment for baseline t-MoCA scores.

Several post hoc sensitivity analyses were performed on the primary and secondary outcomes. The primary outcome was assessed after omitting the MoCA component with the greatest mean change from baseline to 6 to 8 weeks. For the secondary outcome, a complier average causal effect analysis was performed, using 2-stage least-squares instrumental variable regression to account for treatment nonadherence. The first stage of this analysis regressed treatment received on randomly assigned treatment, and the second stage regressed the primary outcome on the predicted probabilities of receiving SENTINEL CEP from the first stage.^4,10,11^ Additional sensitivity analyses included: (1) excluding participants with adjudicated stroke outcomes, (2) separate adjustment for absolute and relative hypotension, (3) multiple imputation of missing MoCA scores among the cohort with a single missing MoCA assessment at either baseline or 6 to 8 weeks, (4) reductions in the total t-MoCA of ≥2 and ≥4 between baseline and 6 to 8 weeks, (5) use of robust standard errors to provide additional protection against variance misspecification, and (6) participants who adhered to assigned treatment allocation using a per-protocol analysis. Multiple imputation by chained equations was used to create 20 imputed datasets, and results were combined using Rubin’s rules. Imputations were performed separately in each treatment group using the following predictors: age, sex, body mass index, hypertension, diabetes, atrial fibrillation, previous stroke or transient ischemic attack, CSHA (Canadian Study of Health and Aging) Clinical Frailty Scale, and t-MoCA scores at baseline, discharge, and 6 to 8 weeks.

Subgroup analyses were performed for the primary and secondary outcome by fitting an interaction between the subgroup and randomized treatment using a generalized linear model. Subgroup analyses were performed for age, sex, baseline cognition (raw MoCA score: ≥26 and <26), baseline dependency (Katz score: independent [6] and not independent [0–5], modified Rankin score: functionally independent [0–2] and functionally independent [3–5]), baseline frailty (CSHA Clinical Frailty Scale: fit [1–3] and vulnerable/frail [4–7]), previous stroke or transient ischemic attack, known dementia or cognitive impairment, other neurological diseases, valve type, the need for predilatation or postdilatation, presence of severe aortic calcification, and the presence of absolute or relative hypotension. In addition, the primary and secondary outcomes were analyzed among participants who had a stroke or transient ischemic attack at 72 hours after TAVI (or hospital discharge if sooner).

All analyses were conducted using Stata software, version 17.0 (StataCorp). Data are presented as mean values with SD or median values with interquartile ranges or counts and percentages. CIs are provided for descriptive purposes only, as analyses were not adjusted for multiplicity.

Deidentified data will be made available to other researchers through the London School of Hygiene and Tropical Medicine Data Compass repository (https://datacompass.lshtm.ac.uk/). These data will be made available subject to completion of a data access agreement. Data will be shared 12 months after the end of the study, which is anticipated to be November 2026 at the earliest.

## RESULTS

### Participant Enrollment

**Figure 1.**
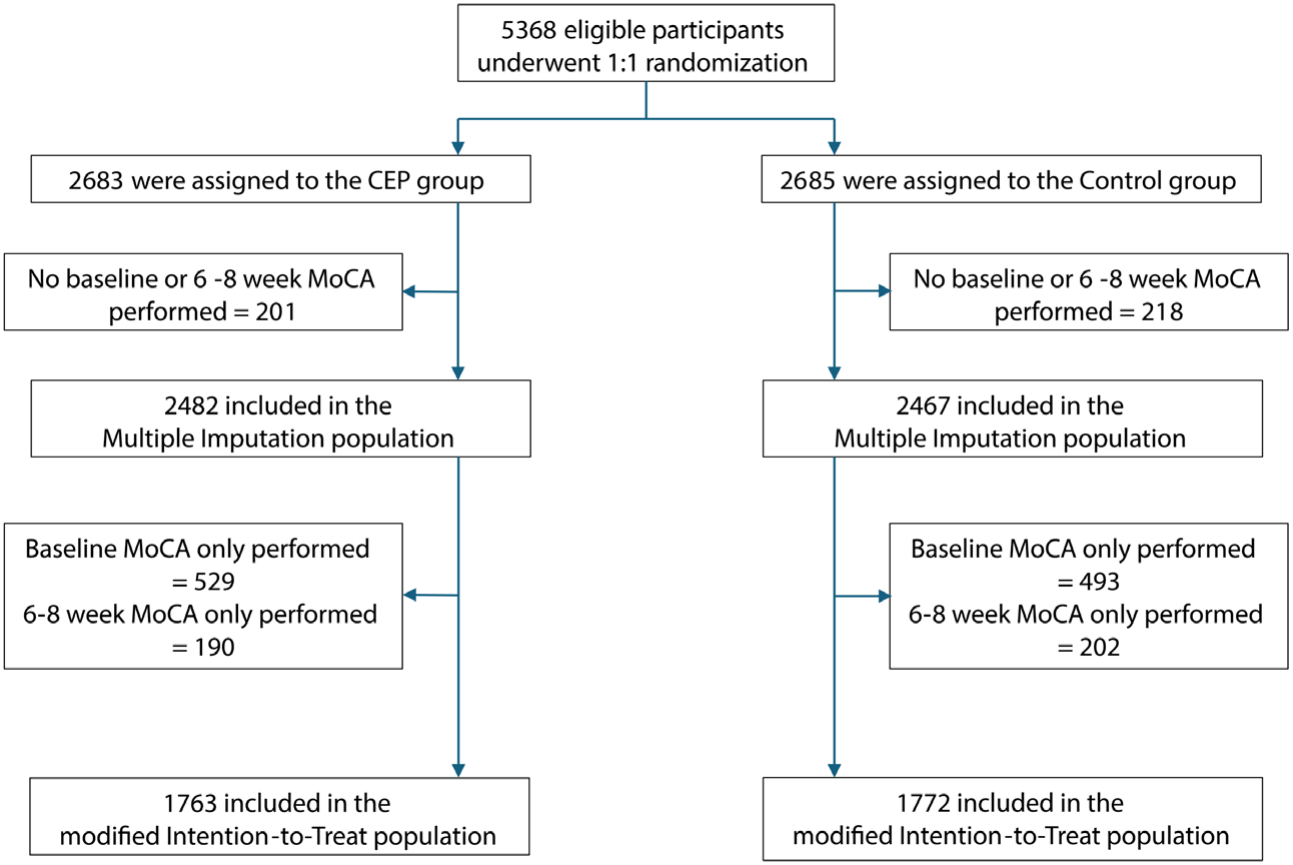
**Flow diagram.** t-MoCA indicates telephone version of the Montreal Cognitive Assessment.

A total of 5368 participants were eligible for this secondary analysis. The modified ITT population comprised 1763 in the SENTINEL CEP group and 1772 in the control group (Figure 1). No MoCA was collected at either baseline or at 6 to 8 weeks on 419 participants. The reasons leading to the MoCA not being collected at baseline and at 6 to 8 weeks follow-up are listed by treatment arm in Table S1. The multiple imputation population included 2482 in the SENTINEL CEP group and 2467 in the control group. Both filters of the SENTINEL CEP device were fully and correctly deployed for the duration of the procedure in 1418 of 1751 participants (81.0%, 12 with missing data) allocated to the SENTINEL CEP group.

The baseline demographics, clinical characteristics, and procedural details of the modified ITT and multiple imputation populations are shown in Table 1 and Table S2, respectively. These were balanced between the groups, apart from the incidence of reported history of coronary artery disease, which was higher in the SENTINEL CEP group (641/1691 [37.9%]) compared with the control group (586/1693 [34.6%], *P*=0.049). The mean age (SD) was 81.0 (6.5) years, and 37.7% of participants were women. As with the main trial, the study cohort was broadly representative of the UK population undergoing TAVI, except that participants from minority ethnic groups were underrepresented (1.7% in this study compared with 4.1% people having TAVI in the United Kingdom). Participants excluded from the modified ITT population were, on average, younger, less likely to have a previous stroke or severe aortic valve calcification and less likely to have a history of congestive heart failure or peripheral vascular disease than those included (Table S3).

**Table 1.**
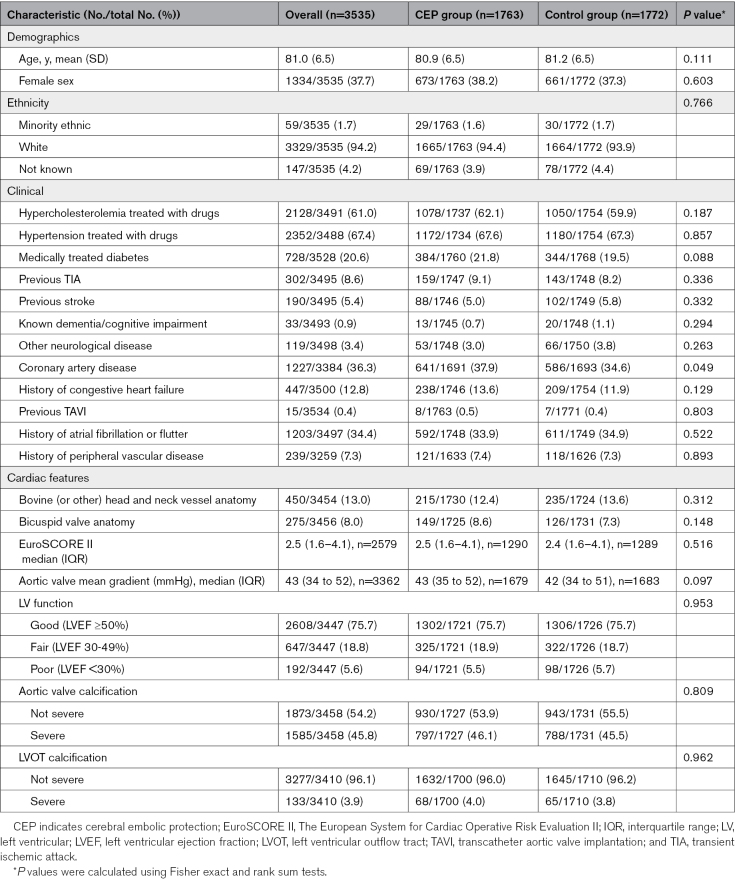
Baseline Demographics and Clinical Characteristics of the Modified Intention-to-Treat Population

The incidence of death within 8 weeks after TAVI was similar in the control (50/2679, 1.9%) and SENTINEL CEP (51/2680, 1.9%) groups.

### Outcomes

**Figure 2.**
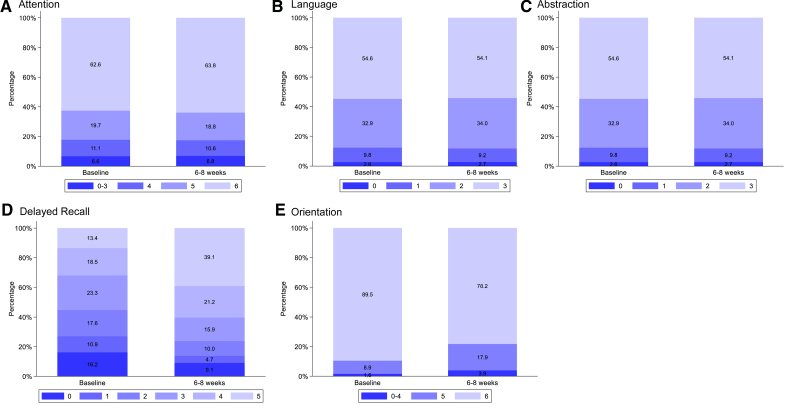
**Telephone MoCA individual component scores.** t-MoCA indicates telephone version of the Montreal Cognitive Assessment.

In the modified ITT population, the median t-MoCA at presentation was 18 (interquartile range, 16–20; Figure S1), and the median t-MoCA at 6 to 8 weeks was 20 (interquartile range, 17–21). The scores for each component of the t-MoCA at baseline and 6 to 8 weeks can be seen in Figure 2. The largest change in the mean score within an individual component was seen with delayed recall, which increased from a mean score of 2.57 at baseline to a mean score of 3.53 at 6 to 8 weeks (*P*<0.0001).

**Figure 3.**
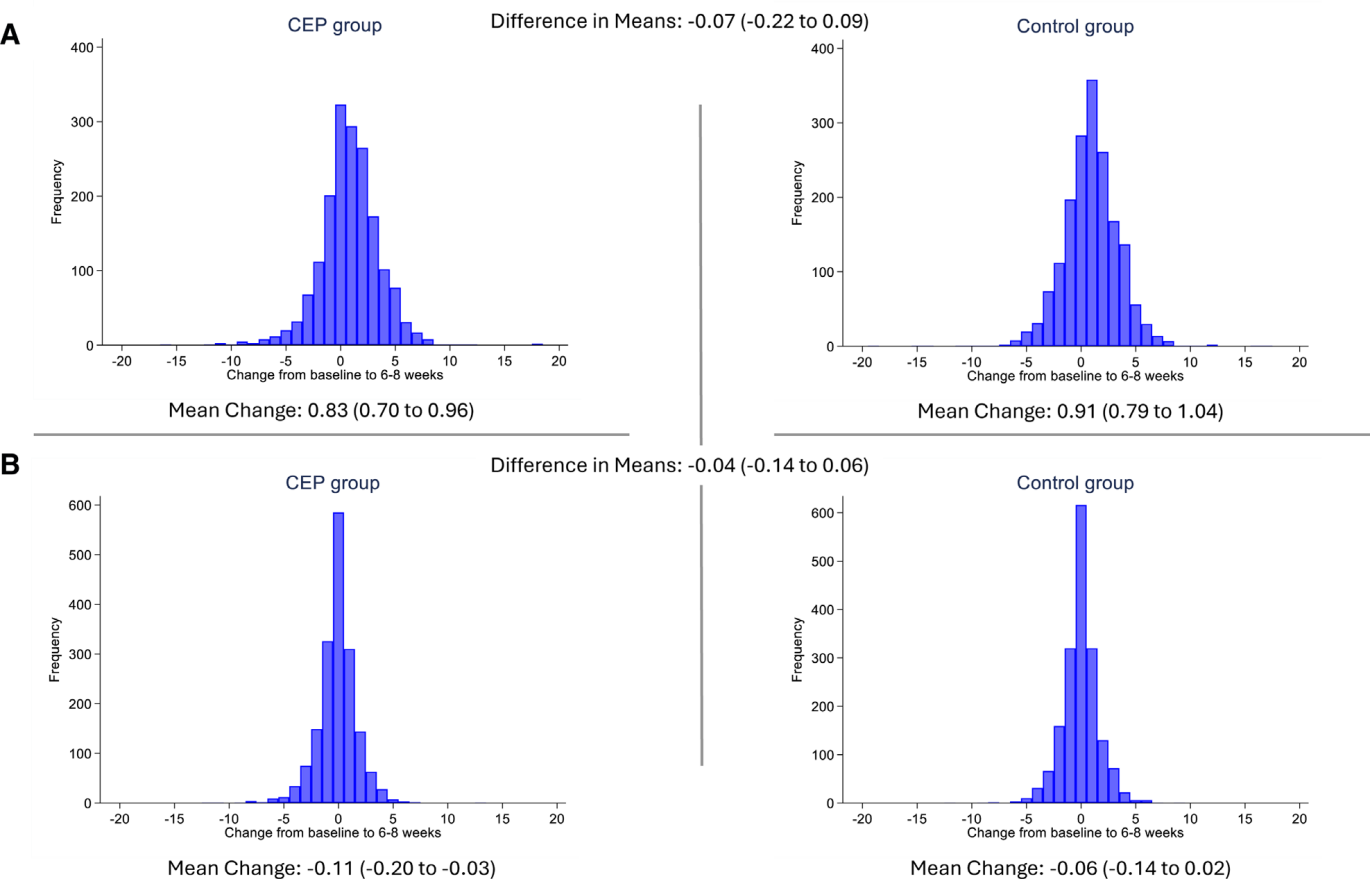
**Primary outcome: distribution of change in total telephone version of the** Montreal Cognitive Assessment **score by treatment arm. A**, Complete telephone version of the Montreal Cognitive Assessment (t-MoCA). **B**, Without delayed recall component score.

The mean change in total t-MoCA score between baseline and 6 to 8 weeks adjusted for the baseline score was 0.83 (95% CI, 0.70–0.96) in the SENTINEL CEP group and 0.91 (95% CI, 0.79–1.04) in the control group. There was no difference in means between the treatment groups (−0.07 [95% CI, −0.22 to 0.09], *P*=0.42; Table 3; Figure 3). In a sensitivity analysis omitting the delayed recall scores, the mean changes from baseline were −0.11 (95% CI, −0.20 to −0.03) in the SENTINEL CEP group and −0.06 (95% CI, −0.14 to 0.02) in the control group. There was no difference in means between the treatment groups (−0.04 [95% CI, −0.14 to 0.06]; Table 2).

**Table 2.**
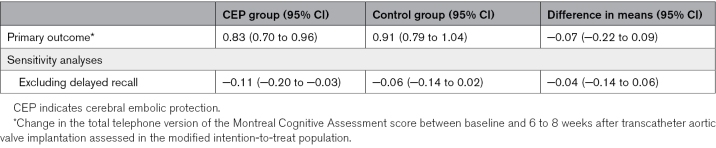
Primary Outcome and Secondary Analyses

**Table 3.**
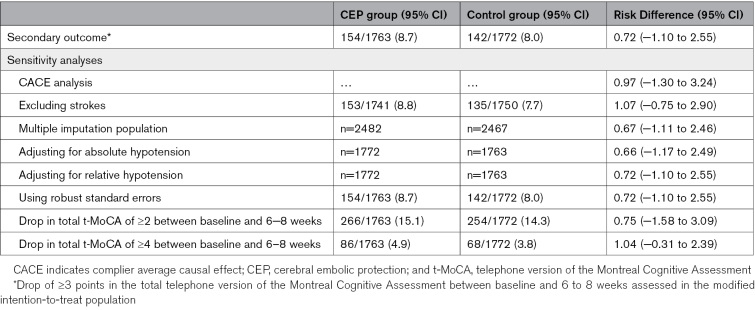
Secondary Outcome and Sensitivity Analyses

In the modified ITT population, the incidence of a ≥3-point drop in the total t-MoCA score between baseline and 6 to 8 weeks was 154 of 1763 (8.7%) in the SENTINEL CEP group and 142 of 1772 (8.0%) in the control group. The corresponding risk difference was 0.72% (95% CI, −1.10 to 2.55; *P*=0.44). The number of adjudicated strokes was 22 in the SENTINEL CEP group and 22 in the control group. Absolute hypotension was present in 857 of 1757 (48.8%) in the SENTINEL CEP group and 780 of 1767 (44.1%) in the control group. Relative hypotension was present in 1128 of 1757 (64.2%) in the SENTINEL CEP group and 1156 of 1767 (65.4%) in the control group. The results of the sensitivity analyses for the secondary outcome are presented in Table 3 and Table S4, and all were consistent with the main analysis of the secondary outcome.

**Figure 4.**
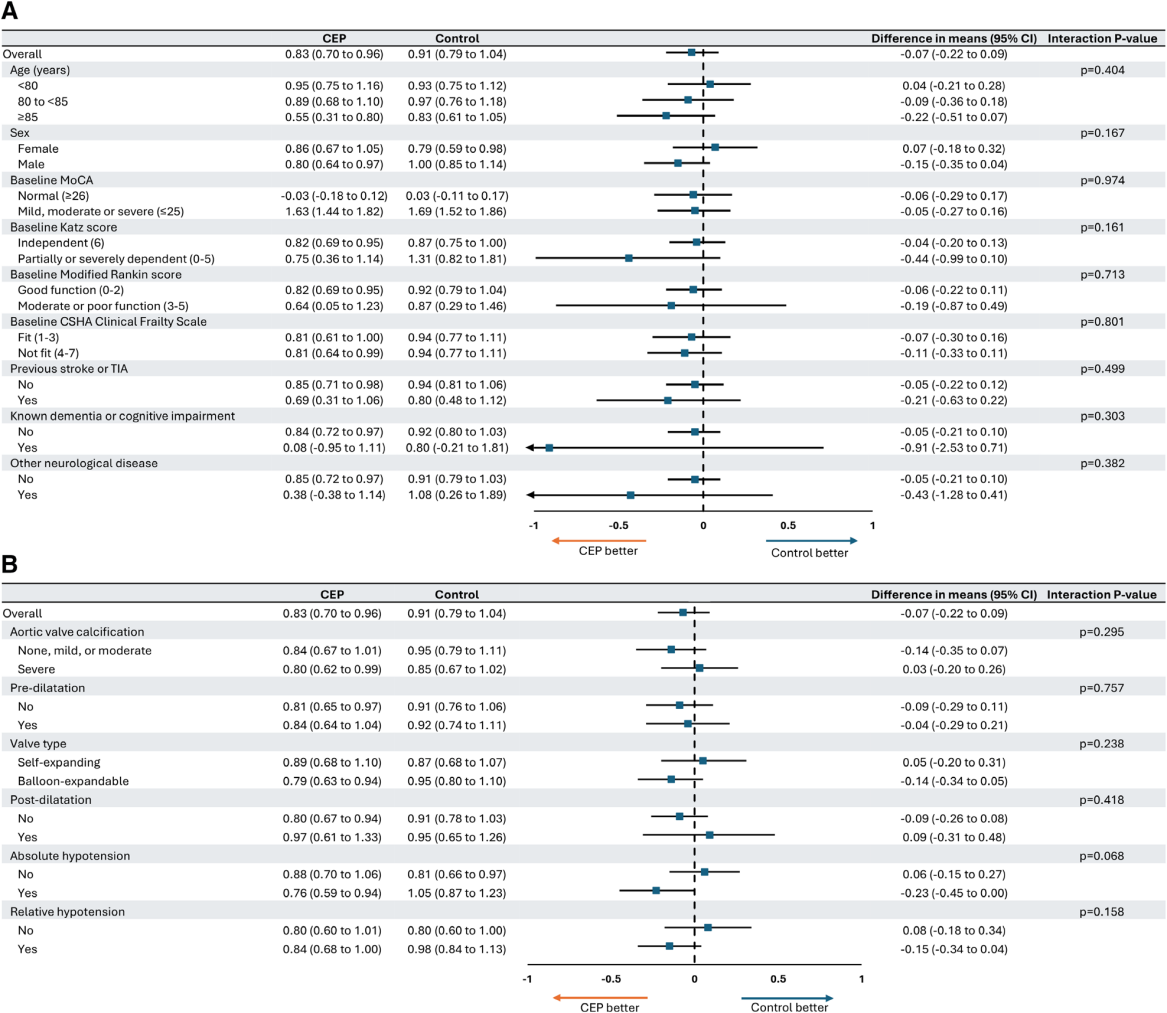
**Mean difference in total telephone version of the** Montreal Cognitive Assessment **score between baseline and 6 to 8 weeks according to subgroup. A**, Patient characteristics. **B**, Procedural characteristics. CEP indicates cerebral embolic protection; CSHA, Canadian Study of Health and Aging; t-MoCA, telephone version of the Montreal Cognitive Assessment; and TIA, transient ischemic attack.

**Figure 5.**
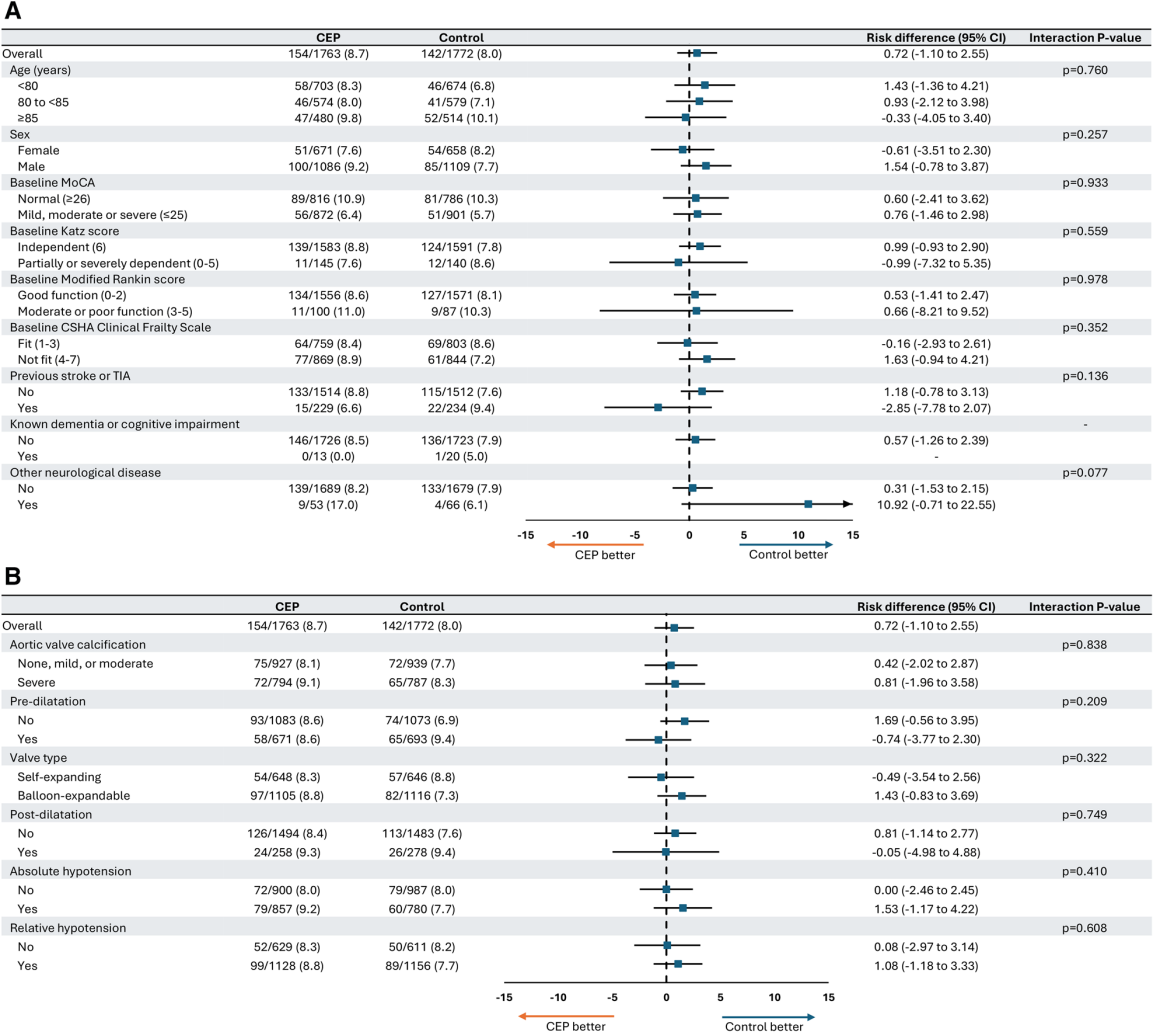
**Incidence of a drop of 3 points or greater in total telephone version of the** Montreal Cognitive Assessment **score between baseline and 6 to 8 weeks according to subgroup. A**, Patient characteristics. **B**, Procedural characteristics. CEP indicates cerebral embolic protection; CSHA, Canadian Study of Health and Aging; t-MoCA, telephone version of the Montreal Cognitive Assessment; and TIA, transient ischemic attack.

Subgroup results for mean difference in total t-MoCA score and a drop of ≥3 in the total t-MoCA score between baseline and 6 to 8 weeks are shown in Figures 4 and 5. There was no evidence of an interaction between treatment assignment and any of the subgroups assessed.

MoCA data were available for 58 participants who had a stroke or transient ischemic attack 72 hours after TAVI (or hospital discharge if sooner) of 194 that occurred in the trial. Consequently, treatment effects for the primary outcome (difference in means, 1.25 [95% CI, −0.25 to 2.74]) and secondary outcome (risk difference, −21.7 [95% CI, −38.9 to −4.4]) among this cohort could not be estimated precisely and should be interpreted with caution (Table S5).

## DISCUSSION

In this secondary analysis of the BHF PROTECT-TAVI trial, we tested the effect of routine CEP on the incidence of cognitive change between baseline and 6 to 8 weeks in patients undergoing TAVI. When using the t-MoCA to assess change in overall cognitive status, there was no treatment effect seen in either the mean change in t-MoCA or when a threshold of a drop of ≥3 in total t-MoCA score between baseline and 6 to 8 weeks was used. Both results were robust to sensitivity analyses. No subgroup was identified as benefitting from the routine use of SENTINEL CEP during TAVI.

The MoCA enabled a simple overall cognitive assessment to be performed at each time point, validated to allow either face-to-face or telephone follow-up, which was essential, given that BHF PROTECT-TAVI recruited participants from across the whole of the United Kingdom. The use of a single MoCA version throughout and the absence of routine magentic resonance imaging for each patient was made to streamline trial procedures, allowing the BHF PROTECT-TAVI trial to maintain its primary focus on assessing the impact of SENTINEL CEP on stroke incidence after TAVI. These choices compromised the ability to explore the granularity of change across multiple cognitive domains (for example, MoCA has limited executive function testing) and the possible relationship of small brain infarcts to cognitive change.^8,12^ However, by the time MoCA data collection stopped, this focused approach had allowed the collection of the MoCA in >3500 participants, a sample size large enough to have a power of >90% to determine a possible treatment effect on overall cognitive status. Even so, we acknowledge that the attrition of participants over time because of death or loss to follow-up may have introduced selection bias.

The choice of using an individual’s change in total t-MoCA score over time as the primary outcome was to avoid the need for population standardization of the MoCA, as the participant would act as their own internal control for their age, education level, and other features that may affect performance during the assessment. No difference was seen in the primary outcome because the mean change in total t-MOCA increased similarly in both treatment arms. Similar improvements in cognitive function after TAVI have been identified before.^13–15^ The reasons for this remain unclear. For instance, the recent CAPTIVA study (Cardiac Output, Cerebral Blood Flow and Cognition in Patients with Severe Aortic Valve Stenosis Undergoing Transcatheter Aortic Valve Implantation) found no association between post-TAVI hemodynamic (cardiac output and cerebral blood flow) and cognitive change.^15^ Given the short time between testing, the identical MoCA version used, and the improvement in the delayed recall component, the increase in mean change in t-MoCA seen in BHF PROTECT-TAVI is most likely attributable to practice effects rather than a true cognitive improvement.^16,17^ The sensitivity analysis removing the delayed recall scores demonstrated no change in either treatment arm in mean t-MoCA scores with tight CIs, supporting previous observations that there is limited impact overall on cognition for most patients undergoing TAVI.^18^

Overall, 8.4% of those undergoing cognitive assessment in BHF PROTECT-TAVI had a 3-point drop in the t-MoCA score. This matches the available evidence.^8^ This rate of cognitive decline is ≈4 times the adjudicated stroke rate seen in the control arm of BHF PROTECT-TAVI. The clinical and health economic implications for participants having this outcome will be explored upon completion of BHF PROTECT-TAVI and by long-term linkage to the UK National Health Service and other data sources.

Numerous tools are available for the assessment of cognitive function, but there is a lack of consensus about the optimal approach.^19^ MoCA is a widely used instrument in clinical studies. Compared with other cognitive screening tools, it is easy to administer and includes cognitive domains that make it effective in the setting of vascular cognitive impairment. The t-MoCA excludes tasks requiring visual or motor responses. One recent study has compared results from face-to-face MoCA 22 with t-MoCA and demonstrated that they are comparable.^20^

In this analysis, we used a change in MoCA score as the primary outcome to detect differences between the assigned treatment groups. However, it is important to consider how changes in MoCA score may be interpreted and the difference between the minimal detectable change at a population level and what may be clinically significant at an individual level. Although there is ongoing debate about the precise threshold, a change of 3 points is generally regarded as a robust threshold change for an individual, beyond normal testing variability.^21–23^ In the primary analysis, small improvements in MoCA score were observed after TAVI, with corresponding CIs that were narrow and excluded differences large enough to be considered clinically significant. When a drop of at least 3 points was assessed, results were consistent with the primary analysis. Both analyses suggest no evidence of a clinically meaningful effect of CEP on cognitive function.

An important point to note, relevant for clinical practice, is that only approximately 1% of participants reported a history of cognitive impairment or dementia to research staff. While acknowledging that cognition was not the primary focus of BHF PROTECT-TAVI and that the baseline full MoCA scores were not population standardized, just >50% of participants in this study had a full MoCA score of ≤25, much more in keeping with previous estimates of the incidence of mild cognitive impairment, at around a third of patients with severe aortic stenosis scheduled for TAVI.^24^ Both patient^25,26^ and physician^27,28^ factors have been implicated in the underreporting of impaired cognition. These findings point to the need to improve the identification of those with mild cognitive impairment in a cardiovascular clinic setting that may help anticipate the risk of post-TAVI complications, such as delirium.^29^

Limitations of this study include the use of a single tool to assess cognitive function, the short timeframe for follow-up assessment, and loss to follow-up of a proportion of participants. Nevertheless, this is one of the largest reported series of MoCA assessments in a TAVI cohort and will inform future work in this area.

In this secondary analysis of the BHF PROTECT-TAVI trial, the use of SENTINEL CEP did not impact cognition, as measured by t-MoCA after TAVI. Most participants’ cognition was unchanged 6 to 8 weeks after TAVI. There was around 4 times the rate of a decline of 3 or more in total t-MoCA as stroke outcomes, pointing to the ongoing need to understand the long-term effects of central nervous system outcomes in patients undergoing TAVI.

## ARTICLE INFORMATION

### Acknowledgments

The authors thank the participants who took part in the BHF PROTECT-TAVI trial.

### Sources of Funding

BHF PROTECT-TAVI is funded by the British Heart Foundation (BHF; clinical study No. CS/20/1/34732). Funding for the CEP devices is provided by Boston Scientific, Inc, which was not involved in the coordination, conduct, or reporting of the study.

### Disclosures

Dr Blackman reports consultant fees from Abbott Vascular, Boston Scientific, Edwards Lifesciences, JenaValve Technology, and Medtronic. Dr Dodd reports funding from the British Heart Foundation (BHF) to work on the BHF PROTECT-TAVI trial. Ms Read reports funding from the National Institute for Health and Care Research (NIHR303450). Ms Jamal, Mr Evans, and Mr Clayton report funding from BHF to work on the BHF PROTECT-TAVI trial. Dr Kharbanda reports an investigator-initiated grant from Boston Scientific for the BHF PROTECT-TAVI trial, speaker fees from Edwards Lifesciences, and speaker fees and advisory board fees from Medtronic. Dr Hildick-Smith reports consultant fees from Abbott Laboratories, Boston Scientific, Edwards Lifesciences, Emboline, Medtronic, Terumo, and W.L Gore & Associates.

### Supplemental Material

Power Calculations Submitted to the Trial Steering Committee Before Discontinuation of MoCA Data Collection

Tables S1–S5

Figure S1

CONSORT Checklist

## Supplementary Material


