# Supplementary material for: Impact of Cerebral Embolic Protection on Cognitive Function After Transcatheter Aortic Valve Implantation: Data From the BHF PROTECT-TAVI Randomized Trial
Source: Circulation. 2025 Aug 30;152(18):1268–78. doi: 10.1161/CIRCULATIONAHA.125.076761 (PMC12577660; doi:10.1161/CIRCULATIONAHA.125.076761)

Supplementary Material:

1) Power Calculations submitted to Trial Steering Committee prior to Discontinuation of MoCA data collection.

2) Supplementary Tables:

Table S1: Reasons why MoCA not completed at baseline and at 6-8 weeks.

Table S2: Baseline Demographics and clinical characteristics of the multiple imputation population.

Table S3: Baseline Demographics and clinical characteristics of participants excluded from the modified intention to treat analysis

Table S4: Primary and secondary outcome in the per-protocol population

Table S5: Subgroup analyses in participants who had a stroke or TIA at 72 hours (or at hospital discharge, if sooner) for the primary and secondary outcome.

3) Supplementary Figures:

Figure S1: Distribution of telephone MoCA scores at baseline

1) **Power Calculations submitted to Trial Steering Committee**

Table A: Sample size calculations for MoCA (90% power) based on a standard deviation of 3.50 (rounded up to the nearest 10)

		Difference to detect in MoCA between treatments		
		**0.5**	**0.75**	**1**
**Correlation**	0	2060	920	520
	0.5	1550	690	390
	0.65	1190	530	300

Table B: Sample size calculations for MoCA (90% power) based on a standard deviation of 3.75

		Difference to detect in MoCA between treatments		
		**0.5**	**0.75**	**1**
**Correlation**	0	2370	1060	600
	0.5	1780	790	450
	0.65	1370	610	350

Table C: Sample size calculations for MoCA (90% power) based on a standard deviation of 4.00

		Difference to detect in MoCA between treatments		
		**0.5**	**0.75**	**1**
**Correlation**	0	2690	1200	680
	0.5	2020	900	510
	0.65	1560	690	390

The correlations above are shown to demonstrate the impact allowing for the correlation between baseline and follow-up has on the sample size.

Table S1: Reasons why MoCA not completed at baseline and at 6-8 weeks.

	**Reason MoCA not completed**	**CEP**	**Control**
**Baseline**	Barrier to communication	24	16
	Environmental/participant factors preventing administration of MoCA	149	173
	Not known	163	149
	Participant declined	57	80
**6-8 weeks follow-up**	Barrier to communication	29	28
	Environmental/participant factors preventing administration of MoCA	13	15
	Lost to follow-up	17	17
	Not known	85	84
	Participant declined	59	70
	Withdrew from the trial		2

Table S2: Baseline Demographics and clinical characteristics of the multiple imputation population.

Characteristic<br>no. / total no. (%)	Overall<br>(N = 4949)	Sentinel CEP Group<br>(N = 2482)	Control group<br>(N = 2467)	P-value*
**Demographics**				
Age (years), mean (SD)	81.3 (6.5)	81.2 (6.5)	81.3 (6.5)	0.274
Female sex (no. / total no. (%))	1894/4949 (38.3)	959/2482 (38.6)	935/2467 (37.9)	0.599
Ethnicity (no. / total no. (%))				0.343
Minority ethnic	81/4949 (1.6)	42/2482 (1.7)	39/2467 (1.6)	
White	4673/4949 (94.4)	2352/2482 (94.8)	2321/2467 (94.1)	
Not known	195/4949 (3.9)	88/2482 (3.5)	107/2467 (4.3)	
**Clinical** (no. / total no. (%))				
Hypercholesterolemia treated with drugs	2991/4867 (61.5)	1537/2440 (63.0)	1454/2427 (59.9)	0.027
Hypertension treated with drugs	3295/4874 (67.6)	1664/2441 (68.2)	1631/2433 (67.0)	0.409
Medically treated diabetes	1021/4942 (20.7)	524/2479 (21.1)	497/2463 (20.2)	0.419
Prior TIA	418/4883 (8.6)	225/2453 (9.2)	193/2430 (7.9)	0.125
Prior stroke	284/4884 (5.8)	139/2453 (5.7)	145/2431 (6.0)	0.669
Known dementia/ cognitive impairment	46/4881 (0.9)	20/2452 (0.8)	26/2429 (1.1)	0.377
Other neurological disease	154/4884 (3.2)	72/2454 (2.9)	82/2430 (3.4)	0.413
Coronary artery disease	1690/4669 (36.2)	885/2349 (37.7)	805/2320 (34.7)	0.036
History of congestive heart failure	680/4901 (13.9)	354/2457 (14.4)	326/2444 (13.3)	0.283
Previous TAVI	21/4947 (0.4)	8/2482 (0.3)	13/2465 (0.5)	0.284
History of atrial fibrillation or flutter	1665/4889 (34.1)	832/2455 (33.9)	833/2434 (34.2)	0.809
History of peripheral vascular disease	354/4524 (7.8)	182/2285 (8.0)	172/2239 (7.7)	0.740
EuroSCORE II<br>median (IQR)	2.6 (1.6 to 4.3),<br>n=3554	2.6 (1.7 to 4.3),<br>n=1774	2.5 (1.6 to 4.2),<br>n=1780	0.463
Bovine (or other) head and neck vessel anatomy (no. / total no. (%))	623/4823 (12.9)	313/2428 (12.9)	310/2395 (12.9)	0.966
Bicuspid valve anatomy<br>(no. / total no. (%))	389/4830 (8.1)	209/2421 (8.6)	180/2409 (7.5)	0.139
Aortic valve mean gradient (mmHg), median (IQR)	43 (34 to 52),<br>n=4687	43 (35 to 52),<br>n=2350	42 (34 to 52),<br>n=2337	0.101
LV function (no. / total no. (%))				0.614
Good (LVEF ≥50%)	3634/4812 (75.5)	1804/2408 (74.9)	1830/2404 (76.1)	
Fair (LVEF 30-49%)	903/4812 (18.8)	462/2408 (19.2)	441/2404 (18.3)	
Poor (LVEF <30%)	275/4812 (5.7)	142/2408 (5.9)	133/2404 (5.5)	
Aortic valve calcification<br>(no. / total no. (%))				0.872
Not severe	2567/4839 (53.0)	1281/2436 (52.6)	1286/2403 (55.5)	
Severe	2272/4839 (47.0)	1155/2436 (47.4)	1117/2403 (46.5)	
LVOT calcification (no. / total no. (%))				0.988
Not severe	4617/4801 (96.2)	2319/2410 (96.2)	2298/2391 (96.1)	
Severe	184/4801 (3.8)	91/2410 (3.8)	93/2391 (3.9)	

Key: CEP = cerebral embolic protection device; IQR = interquartile range; LV = left ventricular; LVEF = left ventricular ejection fraction; LVOT = left ventricular outflow tract; SD = standard deviation; TAVI = transcatheter aortic valve implantation; TIA = transient ischemic attack.

* P-values were calculated using Fisher’s exact and rank sum tests.

Table S3: Baseline Demographics and clinical characteristics of participants excluded from the modified intention to treat analysis

	Included in mITT (n = 3535)	Excluded from mITT (n = 1833)	Overall (n = 5368)	P-value*
**Demographics**				
Age (years), mean (SD)	81.0 (6.5), n=3535	81.8 (6.6), n=1833	81.3 (6.6), n=5368	<0.001
Sex, n/N (%)				0.214
Female	1334/3535 (37.7)	724/1833 (39.5)	2058/5368 (38.3)	
Male	2201/3535 (62.3)	1109/1833 (60.5)	3310/5368 (61.7)	
Ethnicity, n/N (%)				0.131
Asian, black, mixed, or other	59/3535 (1.7)	37/1833 (2.0)	96/5368 (1.8)	
White	3329/3535 (94.2)	1738/1833 (94.8)	5067/5368 (94.4)	
Unknown	147/3535 (4.2)	58/1833 (3.2)	205/5368 (3.8)	
Clinical (no. / total no. (%))				
Hypercholesterolaemia treated with drugs, n/N (%)	2128/3491 (61.0)	1122/1791 (62.6)	3250/5282 (61.5)	0.244
Hypertension treated with drugs, n/N (%)	2352/3488 (67.4)	1204/1797 (67.0)	3556/5285 (67.3)	0.757
Medically treated diabetes, n/N (%)	728/3528 (20.6)	377/1832 (20.6)	1105/5360 (20.6)	0.972
Prior TIA, n/N (%)	302/3495 (8.6)	142/1801 (7.9)	444/5296 (8.4)	0.374
Prior stroke, n/N (%)	190/3495 (5.4)	131/1802 (7.3)	321/5297 (6.1)	0.009
Known dementia or cognitive impairment, n/N (%)	33/3493 (0.9)	21/1798 (1.2)	54/5291 (1.0)	0.471
Other neurological disease, n/N (%)	119/3498 (3.4)	51/1797 (2.8)	170/5295 (3.2)	0.285
Coronary artery disease, n/N (%)	1227/3384 (36.3)	592/1621 (36.5)	1819/5005 (36.3)	0.875
History of congestive heart failure, n/N (%)	447/3500 (12.8)	327/1816 (18.0)	774/5316 (14.6)	<0.001
Previous TAVI, n/N (%)	15/3534 (0.4)	9/1832 (0.5)	24/5366 (0.4)	0.830
History of atrial fibrillation or flutter, n/N (%)	1203/3497 (34.4)	589/1808 (32.6)	1792/5305 (33.8)	0.188
History of peripheral vascular disease, n/N (%)	239/3259 (7.3)	151/1626 (9.3)	390/4885 (8.0)	0.019
Arch vessel anatomy, n/N (%)				0.485
Normal	3004/3454 (87.0)	1557/1776 (87.7)	4561/5230 (87.2)	
Bovine or other	450/3454 (13.0)	219/1776 (12.3)	669/5230 (12.8)	
Native bicuspid valve, n/N (%)				0.957
Bicuspid	275/3456 (8.0)	143/1782 (8.0)	418/5238 (8.0)	
Tricuspid	3181/3456 (92.0)	1639/1782 (92.0)	4820/5238 (92.0)	
Euroscore II, median (IQR)	2.5 (1.6 to 4.1), n=2579	2.7 (1.7 to 4.7), n=1339	2.5 (1.6 to 4.3), n=3918	0.002

Aortic valve mean gradient (mmHg), median (IQR)	43 (34 to 52), n=3362	42 (35 to 53), n=1719	43 (35 to 52), n=5081	0.296
LV function, n/N (%)				0.875
Good (LVEF ≥50%)	2608/3447 (75.7)	1328/1769 (75.1)	3936/5216 (75.5)	
Fair (LVEF 30-49%)	647/3447 (18.8)	338/1769 (19.1)	985/5216 (18.9)	
Poor (LVEF <30%)	192/3447 (5.6)	103/1769 (5.8)	295/5216 (5.7)	
Aortic valve calcification, n/N (%)				<0.001
None	125/3458 (3.6)	79/1796 (4.4)	204/5254 (3.9)	
Mild	598/3458 (17.3)	227/1796 (12.6)	825/5254 (15.7)	
Moderate	1150/3458 (33.3)	585/1796 (32.6)	1735/5254 (33.0)	
Severe	1585/3458 (45.8)	905/1796 (50.4)	2490/5254 (47.4)	
LVOT calcification, n/N (%)				0.865
None	2403/3410 (70.5)	1260/1810 (69.6)	3663/5220 (70.2)	
Mild	625/3410 (18.3)	334/1810 (18.5)	959/5220 (18.4)	
Moderate	249/3410 (7.3)	142/1810 (7.8)	391/5220 (7.5)	
Severe	133/3410 (3.9)	74/1810 (4.1)	207/5220 (4.0)	

Key: CEP = cerebral embolic protection device; IQR = interquartile range; LV = left ventricular; LVEF = left ventricular ejection fraction; LVOT = left ventricular outflow tract; mITT = modified intention to treat; SD = standard deviation; TAVI = transcatheter aortic valve implantation; TIA = transient ischemic attack.

* P-values were calculated using Fisher’s exact and rank sum tests.

Table S4: Primary and secondary outcome in the per-protocol population

	**CEP Group (n=1412)**	**Control Group (n=1761)**	**Difference in means* or risk difference (95% CI)**
Change from baseline to 6-8 weeks, mean (95% CI)	0.82 (0.68 to 0.96)	0.92 (0.80 to 1.05)	-0.06 (-0.23 to 0.11)
Drop of at least 3 points at 6-8 weeks, n/N (%)	117/1412 (8.3)	140/1761 (8.0)	0.34 (-1.58 to 2.25)

* Adjusted for baseline values of the outcome.

Key: CEP = cerebral embolic protection, CI = confidence interval

All participants included in the per-protocol analysis had the TAVI device successfully implanted, and those in the CEP group had to have both filters successfully deployed for the duration of the procedure.

Table S5: Subgroup analyses in participants who had a stroke or TIA at 72 hours (or at hospital discharge, if sooner) for the primary and secondary outcome

	CEP Group (n=30)	Control Group (n=28)	Difference in means* or risk difference (95% CI)
**Change from baseline to 6-8 weeks, mean (95% CI)**	1.33 (0.47 to 2.20)	-0.21 (-1.46 to 1.04)	1.25 (-0.25 to 2.74)
**Drop of at least 3 points at 6-8 weeks, n/N (%)**	1/30 (3.3)	7/28 (25.0)	-21.67 (-38.94 to -4.39)

* Adjusted for baseline values of the outcome.

Key: CEP = cerebral embolic protection, CI = confidence interval

Due to missing data amongst this cohort and small denominators, these data should be interpreted cautiously.

Figure S1: Distribution of t-MoCA scores at baseline

A) Overall

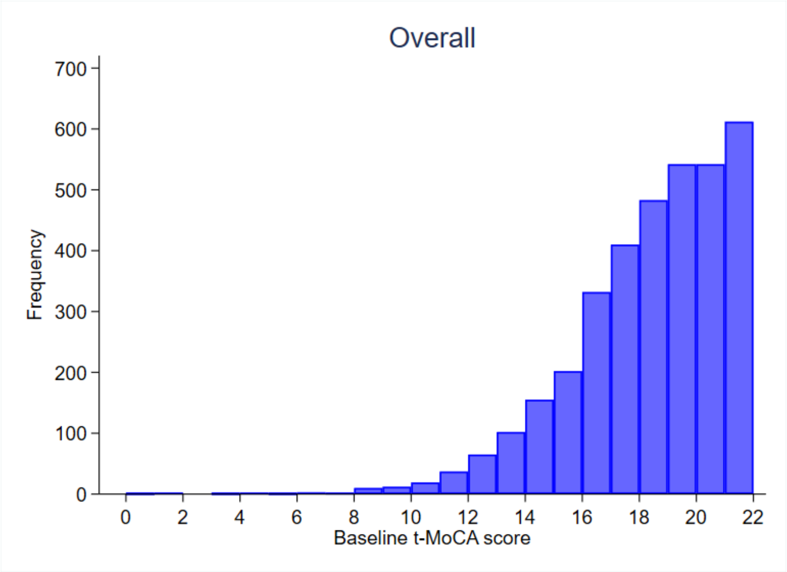

B) Participants assigned to the CEP group

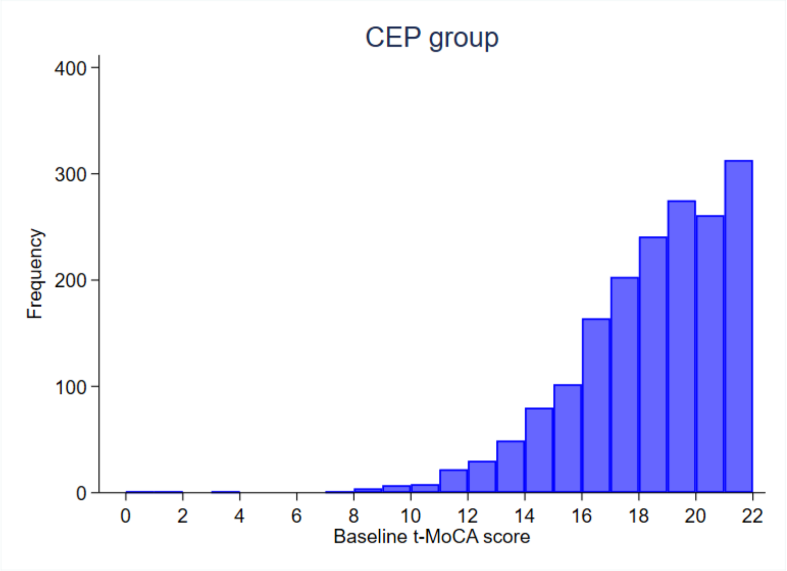

C) Participants assigned to the Control group

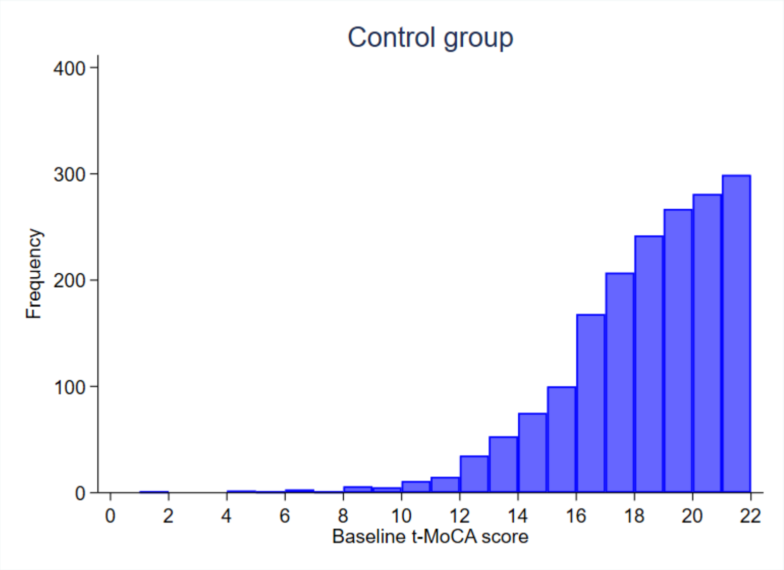

Supplement: Supplementary file 1 [file cir-152-1268-s001.pdf]
